# Some Account of the Effects of the Vapour of Vitriolic Æther in Cases of Phthisis Pulmonalis

**Published:** 1797

**Authors:** Richard Pearson

**Affiliations:** Member of the Royal College of Physicians, London; and Physician to the General Hospital near Birmingham.


X. *Some Account of the Effects of the Vapour of Vitriolic Æther in Cases of Phthisis Pulmonalis. Communicated in a Letter to Dr.* Simmons, *F. R. S. by* Richard Pearson, *M. D. Member of the Royal College of Physicians, London; and Physician to the General Hospital near Birmingham.*

HAVING for the last two years prescribed the vapour of vitriolic æther to patients labouring under phthisis pulmonalis, and having, both in hospital and private practice, experienced the best effects from its use in this frequent and formidable disease, I am preparing to lay before the public a report of the cases in which it has been given, accompanied with remarks on some other remedies that may be employed with advantage in the cure of consumptions. Being desirous, in the recommendation of a new medicine, to have my own evidence supported by the concurrent testimonies of other practitioners, I take the liberty of calling your attention to this subject, and of submit-

ſubmitting to your notice my method of uſing this application, which is ſimply this: I direct the patient to pour one or two tea ſpoonfuls of pure vitriolic æther (or of vitriolic æther impregnated with cicuta, in the manner hereafter deſcribed) into a tea cup or wine glaſs, and afterwards to hold the ſame up to the mouth and draw in the vapour that ariſes from it, with the breath, until all the æther is evaporated. This is repeated three, four, or five times in the courſe of a day, for a month or ſix weeks, more or leſs, according to circumſtances*. The *firſt effects* of this application are

* The loſs of a part of the vapour, which is unavoidable in this mode of applying it, may be prevented, as a medical friend has ſuggeſted, by ſetting the tea cup containing the æther in a ſmall baſon, and inverting a funnel over it. By applying the mouth to the tube of the funnel and making an inſpiration, the patient draws in all the vapour along with the atmoſpheric air, which enters at the bottom of the funnel. In winter, the evaporation may be promoted, by ſetting the tea cup in hot water; in which caſe the funnel is to be inverted, *not* into the baſon containing the water, but over both tea cup and baſon, ſo as to reſt immediately upon a table, tray, or plate, having a bit of doubled paper, or a quill, put under it, to allow the external air to enter more freely.

Children are an agreeable ſenſation of coolneſs in the cheſt, an abatement of the dyſpnœa and cough, and after ten minutes or a quarter of an hour, eaſier expectoration. The *ultimate effects* (provided other proper meaſures be not neglected, for this is not to ſuperſede the uſe of other medicines, but to be employed in conjunction with them) are, a removal of the local inflammation, a cleanſing and healing of the ulcerated lungs, and a ſuppreſſion of the hectic fever. To aſſert that all theſe beneficial conſequences will flow from its application in every ſpecies and degree of phthiſis pulmonalis, would be adopting the language of quacks, and inſulting the underſtanding of every one experienced in the profeſſion; but to ſay that ſome of theſe good effects are likely to reſult from its uſe in moſt inſtances, and moſt of them in a great number of inſtances, is only aſſerting what an experience of two years in a ſitua-

Children and even infants may be made to inhale this vapour, by wetting a handkerchief with æther, and holding it near the noſe and mouth. It muſt be confeſſed, that this is attended with great waſte; but in urgent caſes of hooping cough and croup, in which it promiſes to be of uſe, this conſideration can have little weight.

tion where the opportunities of making trial of it have been very frequent, has fully confirmed.

The ſalutary operation of æther applied to the lungs in the form of vapour, I have found to be greatly promoted by ſeveral volatile ſubſtances that are ſoluble in it; but by none more ſo than by cicuta. By macerating a ſufficient quantity of the dried leaves of this plant in æther for the ſpace of three or four days, or at moſt a week, and occaſionally ſhaking them together, a very ſaturated tincture is obtained, which may be inhaled in the ſame manner, and in the ſame doſes as the pure æther. My proportions are a ſcruple, or half a drachm, of the powdered leaves to every ounce of æther. The narcotic particles of the cicuta, conveyed in this manner along with the æther-vapour to the diſeaſed lungs, act as a topical application, with the beſt effect; hence, æther thus impregnated ſucceeds, in moſt inſtances, better than when it is employed alone. The only unpleaſant circumſtance attending the inhalation of this æthereal tincture of cicuta, is a ſlight degree of ſickneſs and giddineſs, which, however, ſoon go off.

It cannot be expected that I ſhould here point out every ſymptom, or ſet of ſymptoms, which indicate or forbid the uſe of this application. I ſhall only remark, that it appears to be beſt ſuited to the florid, or what is commonly termed the ſcrophulous conſumption. Where the pulmonic affection is complicated with meſenteric obſtruction, or diſeaſes of the other viſcera, or a dropſical condition, it affords but tranſitory relief; and in the very laſt ſtage of the diſorder, the proper time for uſing it is paſt.

Should you be induced, Sir, by this addreſs, to make trial of the vapour of vitriolic æther impregnated with cicuta, in phthiſical caſes, I ſhall be glad to be favoured with your remarks and obſervations upon it, whether in its favour or not.

N. B. In catarrhs, the æther-vapour, without the cicuta, ſucceeds very well. In theſe caſes it is ſeldom neceſſary to continue the inhalation more than three or four days, or a week at fartheſt.

*Birmingham,*

*July* 1. 1796.

